# Effects of the Leptin-Mediated MAPK/ERK Signaling Pathway on Collagen II Expression in Knee Cartilage of Newborn Male Mice from Obese Maternal Offspring

**DOI:** 10.3390/biom12030477

**Published:** 2022-03-21

**Authors:** Wenji Wang, Jialing Zhang, Yu Huo, Yuanzheng Zheng, Yonghao Gui

**Affiliations:** 1 National Children’s Medical Center, Children’s Hospital, Fudan University, Shanghai 201102, China; wenjiwang@fudan.edu.cn (W.W.); zhangjl_61@163.com (J.Z.); 20111240007@fudan.edu.cn (Y.H.); zhengyuanzheng2@sina.com (Y.Z.); 2 National Health Commission (NHC) Key Laboratory of Neonatal Diseases, Fudan University, Shanghai 201102, China; 3 Institute of Pediatrics, Children’s Hospital, Fudan University, Shanghai 201102, China

**Keywords:** cartilage, collagen II, leptin, MAPK/ERK signaling pathway, obese maternal offspring

## Abstract

Epidemiological data suggest that various noncommunicable diseases develop as a result of altered maternal metabolic and physiological status due to exposure to several adverse factors during pregnancy. However, evidence for intrauterine exposure factors and mechanisms underlying the origin of early cartilage disease in chronic osteoarthritic disease is still lacking. In this study, we found that persistent overnutrition during pregnancy in obese mothers led to cartilage damage in neonatal male mice. This was mainly characterized by increased apoptosis with decreased expression of chondrocyte collagen II and low expression of Runx family transcription factor 2 (RUNX2) and SRY-box transcription factor 9 (SOX9). This reduction was also found to be associated with high leptin expression in newborn male mice of obese maternal offspring. Furthermore, the administration of leptin and mitogen-activated protein kinase (MAPK)-extracellular signal-regulated kinase (ERK) inhibitors in primary chondrocytes showed that leptin mediated MAPK/ERK signaling activation and thus affected the key regulators of cartilage matrix metallopeptidase 1 (MMP1) and tissue inhibitor of metalloproteinase 1 (TIMP1), thereby altering the expression of collagen II in mouse cartilage. Altogether, this study provided insights into the molecular mechanisms of cartilage-related disease development and also new clues and evidence for the fetogenetic origin of cartilage diseases.

## 1. Introduction

The “developmental Origins of Health and Disease” theory has upended people’s awareness about disease and health [1]. Adverse exposures early in life may affect lifelong health from childhood to adulthood. A succession of studies in recent years showed that maternal nutritional factors, especially pre-pregnancy and intrapregnancy nutritional deficiencies or overnutrition, could be factors in poor intrauterine exposure, with lasting and profound effects on the cardiovascular, diabetic, and neurocognitive development as well as bone health of the offspring [2,3]. Explorations into the mechanisms of the intrauterine origin of chronic diseases can effectively improve the health of the population and have great scientific, social, and economic value.

The cartilage consists of chondrocytes and the extracellular matrix (ECM) that encases chondrocytes [4]. Under normal physiological circumstances, the cartilage in bone has two primary roles: (1) as a growth plate (or epiphyseal plate), or transitional cartilage, which is an important player in the growth and development of long bones [5], and whose defects dramatically alter the shape and size of the skeleton [6]; and (2) as articular, or permanent cartilage, which maintain the normal functioning of the joint. Chondrocytes are the only functional cells that make up articular cartilage and play an important role in regulating cartilage structure, function, and ECM [7], and the state of chondrocytes during childhood growth and development directly determines the state of long bone health and joint function in adulthood. Osteoarthritis (OA) is the most common degenerative joint disease involving all joint tissues. The pathological changes in OA joints include progressive loss and destruction of articular cartilage, degeneration of cartilage and meniscus, inflammation and fibrosis of the synovial membrane and infrapatellar fat pads, remodeling of subchondral bone, and hypertrophy of the joint capsule [8,9]. Several studies have shown that increased susceptibility to OA can arise from adverse events early in life. For example, exposure to caffeine, ethanol, and dexamethasone during pregnancy has been shown to lead to persistent chondrodysplasia and increased susceptibility to OA in adulthood [10,11,12]. OA continues to affect the health of 13.9% of the population each year and poses a significant disease burden [13]. Exploring the effects of adverse exposure early in life on cartilage health is necessary to gather new clues and evidence for the fetal origin of cartilaginous diseases and the mechanisms of OA.

Over the past decade, overweight and obesity have become serious public health problems in both developed and developing countries [14,15]. Obesity is considered one of the major risk factors for OA in the knees [16,17]. Obese people have high expression of leptin, a hormone secreted by adipose tissue, with growing evidence that leptin is not only a major regulator of energy homeostasis but is also involved in the regulation of glucose homeostasis, neuroendocrine axis, autonomic nervous system, memory, neuroplasticity, and other biological functions [18,19]. In addition, high levels of leptin have been detected in the serum of patients with rheumatoid arthritis and OA, compared with normal individuals [20]. Rodent studies have shown that maternal high-fat diets typically have a negative impact on embryonic skeletal development and bone mass in adult animals [21]. The aforementioned evidence shows (although not clear enough) that obesity, OA, and leptin are biologically related to each other. In addition, the cartilage health of an offspring exposed to maternal obesity and whether leptin mediates chondrogenic diseases and programming mechanisms in one’s offspring during this process are currently unknown.

The mitogen-activated protein kinase (MAPK) signaling pathway is an important pathway that is highly conserved by biological evolution, maintains cellular homeostasis, regulates cellular function, and is activated in response to various stimuli from outside the cell. Evidence from several studies suggests that the MAPK signaling pathway is involved in the development of OA and is an important signaling pathway that regulates cartilage function [22]. In the existing studies, leptin could affect the intervertebral disk cartilage through the MAPK/extracellular signal-regulated kinase (ERK) signaling pathway [23]. Based on this finding, we hypothesized that obesity in mothers would have an impact on the knee cartilage of their offspring and that alterations in leptin-mediated signaling pathways might also be one of the mechanisms involved in the mediation of cartilage health.

Overall, this study explored the effects of maternal obesity on the cartilage health of offspring male mice. The results from an animal model based on a high-fat diet suggested increased apoptosis of knee chondrocytes in newborn male mice of the obese mothers, accompanied by decreased collagen II level, increased serum leptin level, MAPK/ERK signaling pathway activation, and aberrant expression of the cartilage-important regulators SRY-box transcription factor 9 (SOX9) and Runx family transcription factor 2 (RUNX2). Furthermore, in combination with in vitro experiments, we found that the reduction of collagen II in cartilage was closely associated with the leptin-activated MAPK/ERK signaling pathway. The signaling pathways involved and the expression of key molecules provided new evidence and perspectives on cartilage diseases and the mechanisms of the developmental origin of adult osteoarthritis

## 2. Materials and Methods

### 2.1. Animal Model

All procedures and animal handlings complied with the guidelines of the Shanghai Medical Experimental Animal Care and per the 3R-principle under the Animal Experiment Committee of the Children’s Hospital of Fudan University (no. 151-2021). A total of 16 female and 16 male C57BL/6J mice between 4 and 5 weeks of age were obtained from Shanghai SLACCAS Experimental Animal Ltd. (Shanghai, China). The animals were housed at room temperature under a light–dark cycle (12 h light and 12 h dark). After a week of adjustable feeding, female mice were randomly divided into the control group (*n* = 8) and the obese group (*n* = 8). The mice in all groups were fed ad libitum. The control group was fed a normal diet (18.2 kcal% fat, energy 3.5 kcal/g, P1103F-25, SLACOM, Shanghai, China) and given autoclaved distilled water. The obese group was fed a Western diet (41 kcal% fat, energy 4.7 kcal/g, D12079B, Research Diets, New Brunswick, NJ, USA) supplemented with a 20% sucrose solution. Vitamins and minerals were added to the diet of both the normal and obese groups, which was molded as described previously [24]. After 8 weeks on a diet, each female mouse was mated with one male mouse. A total of 25 offspring male mice from the normal diet group and 19 offspring male mice from the obese group were collected. The mice were sacrificed by decapitation on the 7th day after birth and by inhalation of isoflurane at the 1st and 3rd months after birth, respectively. Bilateral cartilage tissues of knee joints were taken from the sacrificed mice for subsequent experimental studies. The plasma leptin and cartilage collagen II expression and associated factor alterations were more obvious in male neonatal offspring mice than in females at the beginning of the experiment (results shown in Supplementary Figure S1B,C), Therefore, offspring male mice were selected for exploring the mechanisms of leptin-mediated alterations in cartilage collagen II and cartilage-important factors in obese offspring in subsequent experiments.

### 2.2. Histological Analysis

After the mice were sacrificed, the knee cartilage specimens were fixed with 4% paraformaldehyde for 24 h and decalcified in 10% EDTA decalcifying solution. Paraffin-embedded tissues were cut into 4 μm sections. Safranin O/Fast Green FCF Cartilage Stain Kit (Solarbio Science & Technology, Beijing, China) and toluidine blue (Beyotime Biotechnology, Shanghai, China) were used to assess imageology and histomorphology differences of the mouse knee joint. Knee cartilage was scored using the OARSI recommended scoring system for osteoarthritis in mice [25], after staining with Safranin O/fast green FCF.

### 2.3. Immunohistochemistry

Mouse knee joints were obtained postmortem and further fixed with 4% paraformaldehyde for 24 h after removing the muscle tissue with scissors. Following fixation, the joints were decalcified (14 days, 20% EDTA, pH 7.4) and embedded in paraffin. Paraffin blocks were cut into slices of 4 μm of thickness. After dewaxing and rehydration, the sections were subjected to pepsin pretreatment for antigen exposure (1 mg/mL of pepsin, 20 min, room temperature). A 5% bovine serum albumin (BSA) was used to block nonspecific antigens. The sections were incubated with collagen II (1:200, ab34712, Abcam) primary antibody overnight at 4 °C and with secondary antibodies for 1 h at room temperature. The images were collected using Pannoramic DESK (3DHISTECH, Budapest, Hungary). Collagen II expression levels are expressed as mean optical density (MOD). MOD = (IOD)/(area SUM). IOD: integrated optical density, which is the cumulative optical density of positive expression in all selected areas in the image. Area SUM: the total area of all selected areas in the image.

### 2.4. Elisa Assay

Newborn day 7 mice were sacrificed by decapitation, and blood was collected into tubes containing sodium heparin. Mouse blood was centrifuged at 2000× *g* for 20 min and plasma was extracted. The detection of plasma leptin was performed according to the steps of the ELISA kit (CRYSTAL CHEM, cat. No. #90030, Downers Grove, IL, USA) instructions.

### 2.5. TdT-Mediated dUTP Nick End Labeling (TUNEL) Staining

The TUNEL kit (GDP1042, Servicebio, Wuhan, China) was used to identify apoptotic chondrocyte DNA fragmentation following the manufacturer’s protocols, and the nuclei were stained with DAPI. The slides were scanned using a Pannoramic digital slide scanner (Pannoramic DESK, 3DHISTECH, Budapest, Hungary). Five fields per slide were randomly selected to quantify TUNEL staining and captured under CaseViewer version 2.0 (3DHISTECH, Budapest, Hungary) software to count TUNEL-positive cells.

### 2.6. Transmission Electron Microscopy

The knee cartilage tissue was rapidly modified cut into 1 mm^3^ tissue blocks and fixed with 2.5% glutaraldehyde for 24 h. Subsequently, the tissues were postfixed, dehydrated at room temperature, infiltrated and embedded, polymerized, ultrathin sectioned, stained, and then observed under a transmission electron microscope (HT7800, Hitachi High Technology Co., Tokyo, Japan).

### 2.7. Cell Culture

Primary chondrocytes were extracted from mice as previously described [26]. Newborn suckling C57BL/6 mice (2–3 days) were obtained from Shanghai JSJ Laboratory Animal Co., Ltd. (Shanghai, China). In brief, after the pups were sacrificed by decapitation, their knee joints were exposed, excess tissue from the surrounding tissues was removed, and hyaline cartilage was dissected out from the cartilage surface. After 0.5 h of trypsin and 3 h of type II collagenase digestion, the cell suspensions were filtered through a 20 µm stainless steel mesh and centrifuged at 1500 rpm for 5 min. The chondrocytes were cultured in DMEM (D6429, Sigma-Aldrich, Burlington, MA, USA) supplemented with 10% fetal bovine serum (FBS) (Ausbian, Sydney, Australia) and 0.5% penicillin/streptomycin (Beyotime Biotechnology, Shanghai, China) and incubated at 37 °C in the presence of 5% CO_2_. Media were changed every 2 days. Leptin (Mouse, HY-P70704, MCE, Monmouth Junction, NJ, USA) and ERK1/2 inhibitor (*PD98059*, HY-12028, MCE, Monmouth Junction, NJ, USA) were given at the appropriate dose and time in the third generation of cells.

### 2.8. Immunofluorescence

The chondrocytes were fixed with 4% paraformaldehyde (Solarbio Science & Technology, Beijing, China). Then, the cells were washed three times and permeabilized with 1% Triton X-100 in PBS for 15 min. The cells were incubated with primary antibody (Collagen II, 1:200, ab34712; Abcam, Cambridge, UK) at 4 °C overnight and washed three times before incubating with a secondary antibody (goat anti-rat Cy3; Jackson ImmunoResearch, Philadelphia, PA, USA) for 1 h. Finally, the cells were incubated with DAPI-containing antifluorescence quenching tablets (Beyotime Biotechnology, Shanghai, China). Microphotographs were captured using a confocal microscope (Leica TCS Sp8; Leica, Wetzlar, Germany).

### 2.9. Flow Cytometry for Detecting Cell Apoptosis

Annexin V-FITC/propidium iodide (PI) Cell Apoptosis Analysis Kit (BD, 556547, San Jose, CA, USA) was used for apoptosis assays with a flow cytometer (BD FACSCelesta, San Jose, CA, USA). After the administration of different doses of leptin for 24 h, the chondrocytes were collected using trypsin without EDTA and washed twice with cold PBS. Then, they were resuspended in 1× binding buffer, followed by mixing with Annexin V and PI at room temperature in the dark for 15 min. The experiments were repeated independently three times. The data were analyzed using FlowJo (version 10, Ashland, Oregon, USA) software.

### 2.10. RNA Extraction and Real-Time RT-PCR

Total RNA from chondrocytes was extracted using TRIzol reagent (Ambion, Life Technologies, Carlsbad, CA, USA) and cDNA was synthesized using *Evo M-MLV* RT Mix Kit with gDNA Clean for qPCR (AG11728; China) following the manufacturer’s protocols. RT-PCR was performed using Light Cycler 480 Real-Time PCR system (Roche Applied Science, Basel, Switzerland) with an SYBR Green Premix Pro Taq HS qPCR kit (AG11701; Accurate Biotechnology, Hunan, China) for mRNA quantitation of all referred genes. The reaction was performed at 95 °C for 30 s, 95 °C for 5 s, and 60 °C for 30 s for 40 cycles. The sequences of all the primers are depicted in Table 1. All genes were normalized to housekeeping gene GAPDH. The changes in the transcript levels of targeted genes were determined using the 2^−ΔΔCT^ method.

### 2.11. Protein Extraction and Western Blot Analysis

The knee cartilage in neonatal mice from these obese mothers on day 7 was homogenized in the RIPA buffer (supplemented with a 1% proteinase and phosphatase inhibitor mixture). The nuclear protein was extracted using a nuclear protein extraction kit (Beyotime Biotechnology, Shanghai, China). The protein concentration of the supernatant was evaluated using bicinchoninic acid (BCA kit, Beyotime Biotechnology, Shanghai, China). Equal amounts of proteins (20–35 μg) were size-fractionated by 8–12% SDS-PAGE and transferred to 0.22 μm polyvinylidene fluoride membranes (Millipore Corporation, MA, USA) using a wet transfer system. The membranes were blocked with 5% BSA for 1 h and probed with primary antibodies against Collagen II (1:1000, ab34712, Abcam), SOX9 (1:1000, AF6330, Affinity), RUNX2 (1:1000, AF5186, Affinity), MMP13 (1:1000, DF6494, Affinity), TIMP1 (1:1000, AF7007, Affinity), MMP1 (1:1000, 10371-2-AP, Proteintech), Leptin-R (1:1000, DF7139, Affinity), Phospho-p44/42 MAPK (Erk1/2) (Thr202/Tyr204) (1:1000, 4370s, CST), Phospho-SAPK/JNK (Thr183/Tyr185) (1:500, 4668, CST), Phospho-p38 MAPK (Thr180/Tyr182) (1:500, 4511, CST), β-actin (1:1000, 4970, CST) and Histone H3 (1:1000, AF0863, Affinity) at 4 °C overnight. After three washes with TBS-T, the filters were incubated with the corresponding HRP-conjugated secondary antibodies (1:2500, 7074, CST) for 1 h at room temperature and protein bands visualized using a Tanon GIS-2020 imaging system (Tanon Science & Technology, Shanghai, China). Image Lab software was used to analyze the bands.

### 2.12. Statistical Analysis

SPSS software (Version 26.0, IBM, Chicago, IL, USA) was used for statistical description and analysis. The Student *t*-test was performed for normally distributed continuous variables or the Wilcoxon rank-sum test for non-normally distributed variables to compare two groups of variables. One-way analysis of variance (ANOVA) was used to compare the difference among groups for variables that had a normal distribution and homogeneous variance. The Kruskal-Wallis H test was used to compare groups for variables that were not normally distributed or had a heterogeneous variance for comparison of more than two groups of variables. Bonferroni post hoc tests were used to test significant comparisons. The correlation between leptin receptor and genes associated with cartilage health mRNA expression comparisons was based on Pearson’s correlation analysis. All reported significance levels were two-sided, and the threshold of statistical significance was *p* < 0.05.

## 3. Results

### 3.1. Effect of Maternal Obesity on Baseline and Knee Cartilage in the Offspring of Neonatal 7-Day-Old Male Mice

**Figure 1:**
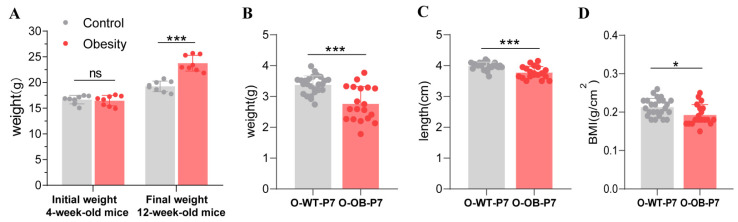
Effect of maternal obesity on offspring 7-day male mice at baseline. (**A**) Changes in body weight in mother after being fed a normal diet and high-fat diet. (**B**) Bodyweight, (**C**) body length, and (**D**) BMI of offspring 7-day male mice in the control and obese groups. O-WT-P7: offspring 7-day male mice from the control mothers (*n* = 25). O-OB-P7: offspring 7-day male mice from the obese mothers (*n* = 19). BMI: body mass index. Results are expressed as mean ± SD. ns not significant, * *p* < 0.05, *** *p* < 0.001.

Sixteen female mice were randomized into two groups of eight mice each and fed either the normal or high-fat diet for 8 weeks. Then, they were mated with normal male mice. After the progeny mice followed the specified diet, the mice in the obese group weighed 20% more than those in the control group, which was the criterion for the successful construction of the obesity model (Figure 1A). Furthermore, we obtained 25 offspring 7-day male mice from the control group (O-WT-P7) and 19 offspring 7-day male mice from the obese group (O-OB-P7). Newborn male mice from the obese group were found to have lower body weight (Figure 1B, *p* < 0.001), body length (Figure 1C, *p* < 0.001), and BMI (Figure 1D, *p* < 0.05) compared with those from the control group.

**Figure 2:**
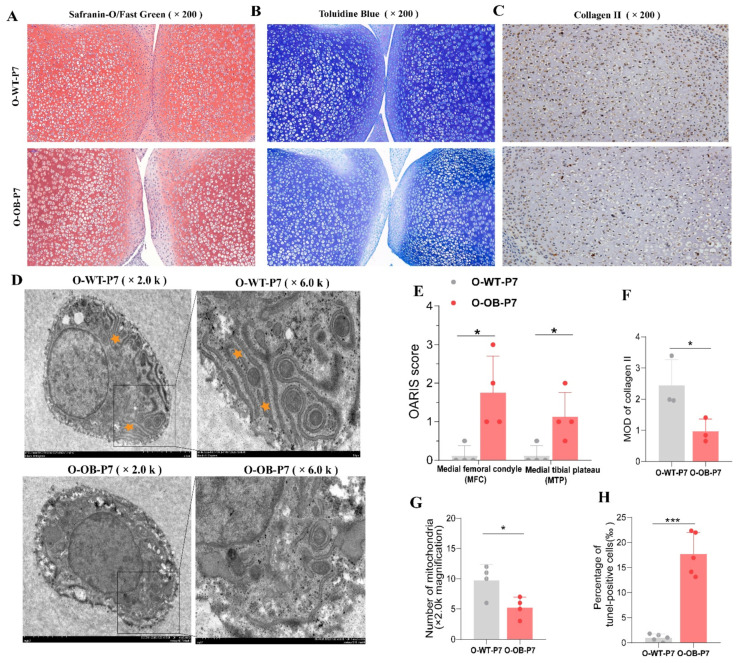
Effect of maternal obesity on knee cartilage in the offspring 7-day male mice. (**A**) Representative images of knee cartilage stained with Safranin O/Fast Green and (**B**) toluidine blue in control versus obese offspring (×200 magnification, *n* = 4). (**C**) Representative images of IHC-stained in collagen II knee cartilage in control versus obese offspring (×200 magnification, *n* = 3). (**D**) Ultrastructural observation of knee chondrocytes in control versus obese offspring (*n* = 4). (**E**) Statistical results of the OARIS scores of knee cartilage in the offspring of each group. (**F**) Statistical results of IHC-stained collagen II in each group. (**G**) Statistical results of mitochondrial count in chondrocytes by transmission electron microscopy (*n* = 4). (**H**) Statistical results of the positive rate of TUNEL staining of knee cartilage in each group (*n* = 5). O-WT-P7: offspring 7-day male mice from the control mothers. O-OB-P7: offspring 7-day male mice from the obese mothers. Yellow stars mean developed endoplasmic reticulum. Results are expressed as mean ± SD. * *p* < 0.05, *** *p* < 0.001.

Knee cartilage of 7-day neonatal male mice from control and obese groups were stained with Safranin O/fast green as well as toluidine blue to examine the health of the cartilage matrix and the results are shown in Figure 2. The results showed that the male mice of the obese group showed uneven staining of the cartilage with Safranin O and lighter staining of the articular surface of the knee cartilage (Figure 2A). In addition, heterogeneous blue staining of knee cartilage was observed with toluidine blue staining, with superficial layers of knee cartilage from obese progeny showing lighter staining (Figure 2B). Bilateral OARIS scores were higher in the knee joints of male neonatal mice from obese offspring than in control offspring (Figure 2E, both *p* < 0.05). This indicated that the cartilage matrix of offspring 7-day male mice of the obese group behaved abnormally relative to that in the control group. Furthermore, we performed collagen II staining using immunohistochemistry (IHC) on knee cartilage in both groups and showed that the expression of cartilage knee collagen II in newborn male mice of the obese group was lower than that in the control group (Figure 2C,F, *p* < 0.05). Next, transmission electron microscopy showed the morphology of chondrocytes in both groups. The knee chondrocytes of male offspring in the control group had clear margins and a well-developed endoplasmic reticulum (Figure 2D). In contrast, the edges of the knee chondrocytes of newborn male mice in the obese group were blurred, accompanied by a decrease in the number of mitochondria and endoplasmic reticulum. Finally, TUNEL staining was performed to detect the apoptosis of chondrocytes in the cartilage of both offspring mice groups. The results showed that the proportion of apoptosis was higher in the obese group than in the control group (Figure 2H, Supplementary Figure S1A, *p* < 0.001). The results indicated that the chondrocytes in the knee cartilage of offspring 7-day male mice in the obese group showed organelle alterations accompanied by increased cell apoptosis and inhomogeneity of the matrix fraction of the articular cartilage surface relative to those in the control group.

### 3.2. Alterations in Important Indicators Associated with Knee Cartilage in Neonatal 7-Day Male Mice from Maternal Obesity Group

**Figure 3:**
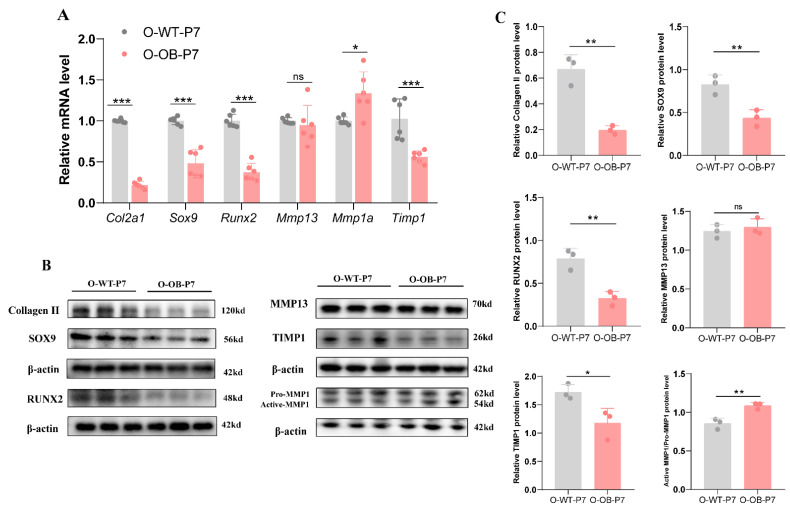
Key indicators expression of knee cartilage in 7-day male mice offspring of control and obese groups. (**A**) Relative mRNA, (**B**) representative western blot bands, and (**C**) statistical results of knee cartilage 7-day male mice offspring in the control group versus obese group. O-WT-P7: offspring 7-day male mice from the control mothers. O-OB-P7: offspring 7-day male mice from the obese mothers. (**A**) *n* = 6 represents six mice in each group from at least three litters of different mothers. (**B**) *n* = 6, each band represents the expression of cartilage tissue proteins in two mice from at least three litters of different mothers. Results are expressed as mean ± SD. ns not significant, * *p* < 0.05, ** *p* < 0.01, and *** *p* < 0.001.

The cartilage ECM plays a vital role in the health of chondrocytes. The cause of decreased collagen II in knee cartilage of the 7-day male mouse offspring in the maternal obesity group was investigated by examining the collagen II-related transcription factors RUNX2 and SOX9 at the mRNA and protein levels. The results showed that the expression of RUNX2 and SOX9 mRNA (Figure 3A, both *p* < 0.001) and protein levels (Figure 3B,C, both *p* < 0.01) in the cartilage of the obese group offspring was significantly downregulated relative to the control group.

Second, we examined the mRNA and protein levels of matrix metallopeptidase 13 (MMP13), MMP1, and tissue inhibitor of metalloproteinase 1 (TIMP1), which are associated with collagen II degradation. The results showed no statistically significant differences in the expression of MMP13 in the two groups at the mRNA and protein levels (Figure 3A–C, both *p* > 0.05). *Mmp1a* is a functional *MMP1* homologue [27]. MMP1a mRNA and protein levels were elevated in the knee cartilage of newborn male 7-day mice in the maternal obesity group relative to those in the control group (Figure 3A, *p* < 0.05, Figure 3B,C, *p* < 0.01, respectively), while TIMP1 mRNA and protein expression levels decreased (Figure 3A, *p* < 0.001, Figure 3B,C, *p* < 0.05, respectively).

The aforementioned experiments demonstrated that reduced collagen II in the cartilage of male neonatal mice of obese mothers might be associated with the decreased levels of RUNX2 and SOX9 and also with the elevated expression of MMP1 and reduced expression of TIMP1, further adding to the evidence of cartilage matrix damage in male neonatal mice in the maternal obesity group.

### 3.3. Leptin Signaling Pathway (MAPK/ERK) Was Activated in the Knee Cartilage of Male Neonatal Mice from the Maternal Obesity Group

**Figure 4:**
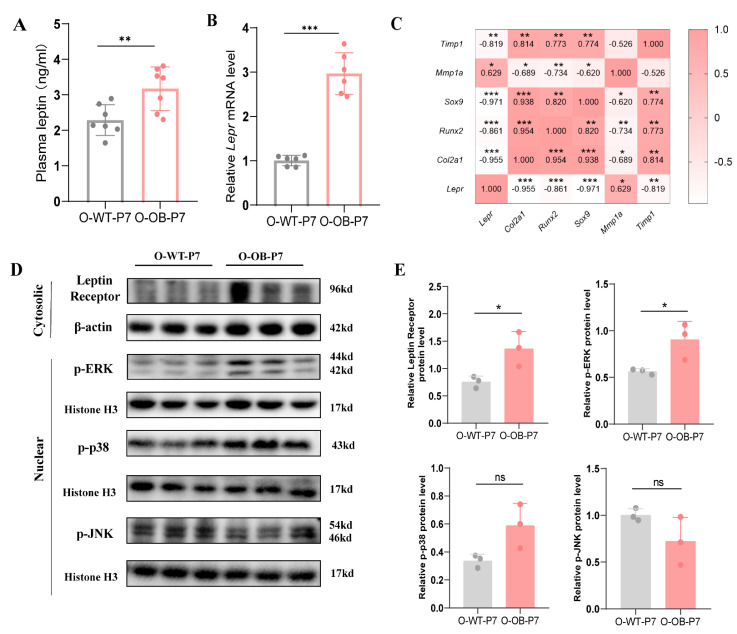
Leptin signaling pathway (MAPK/ERK) was activated in the knee cartilage of 7-day male mice from the maternal obesity group. (**A**) Plasma leptin level and (**B**) *Lepr* mRNA level of knee cartilage 7-day male mice offspring in control versus obesity groups. (**C**) Correlation between *Lepr* and cartilage key indicators mRNA expression levels. (**D**) Representative western blot bands of the expression of leptin receptor in the cell cytoplasm and phosphorylated ERK (p-ERK), phosphorylated p38 (p-38), and phosphorylated c-Jun N-terminal kinase (p-JNK) in the nucleus from knee cartilage of the offspring. (**E**) Statistical results of the expression of key proteins of MAPK signaling pathway in knee cartilage of the offspring. (**A**) *n* = 7, (**B**) *n* = 6, (**C**) *n* = 6, and (**D**) *n* = 6; each band represents the expression of cartilage tissue proteins in two mice. All mice in each group from at least three litters of different mothers. O-WT-P7: offspring 7-day male mice from the control mothers. O-OB-P7: offspring 7-day male mice from obese mothers. Results are expressed as mean ± SD. ns not significant, * *p* < 0.05, ** *p* < 0.01, and *** *p* < 0.001.

Obesity has been known to induce adipokines to release more leptin, which, in turn, is involved in the disease progression of OA [20]. Therefore, we measured the plasma levels of leptin in neonatal 7-day male mice from the control group versus the maternal obesity group using ELISA. The results showed higher plasma leptin levels in newborn male mice from the maternal obesity group than in the control group (Figure 4A, *p* < 0.01). In addition, *Lepr* mRNA levels were higher in the cartilage of male neonatal mice from the maternal obesity group than in the control group (Figure 4B, *p* < 0.001).

Leptin signaling through leptin receptors can be mediated via the MAPK pathway. Therefore, we examined the expression of key proteins of the cartilage MAPK signaling pathway in the cytoplasm and nucleus of both groups. As depicted in Figure 4D, the levels of leptin receptor and p-ERK were significantly higher in the maternal obesity group than in the control group (Figure 4E, both *p* < 0.05). However, no significant differences were detected in p-p38, p-JNK protein levels (Figure 4E, both *p* > 0.05).

Whether leptin is potentially related to abnormal expression of key cartilage genes was investigated by correlating *Lepr* with mRNA expression of chondrogenic key regulators, and the results are shown in Figure 4C. The *Lepr* expression negatively correlated with *Col2a1* expression in cartilage (*r* = −0.955, *p* < 0.001), negatively correlated with the transcription factor *Sox9* and *Runx2* expression (*r* = −0.971, *p* < 0.001; *r* = −0.861, *p* < 0.001), positively correlated with the expression of *Mmp1a* (*r* = 0.629, *p* < 0.05), and negatively correlated with the expression of *Timp1* (*r* = −0.819, *p* < 0.01).

The aforementioned results suggested that leptin expression increased in male neonatal mice from the maternal obesity group, and the MAPK/ERK signaling pathway was activated in the cartilage of male neonatal mice from the maternal obesity group. In addition, at the transcriptional level, *Lepr* expression appeared to be correlated with key regulators of cartilage.

### 3.4. Expression of Key Cartilage-Related Indicators under the Effect of Different Doses of Leptin In Vitro Experiments

To further demonstrate whether leptin could mediate the alterations in key regulators of chondrocytes, we utilized primary mouse chondrocytes cultured in vitro and observed expression at the level of chondrogenically important regulatory transcripts and proteins under different doses of leptin administration.

**Figure 5:**
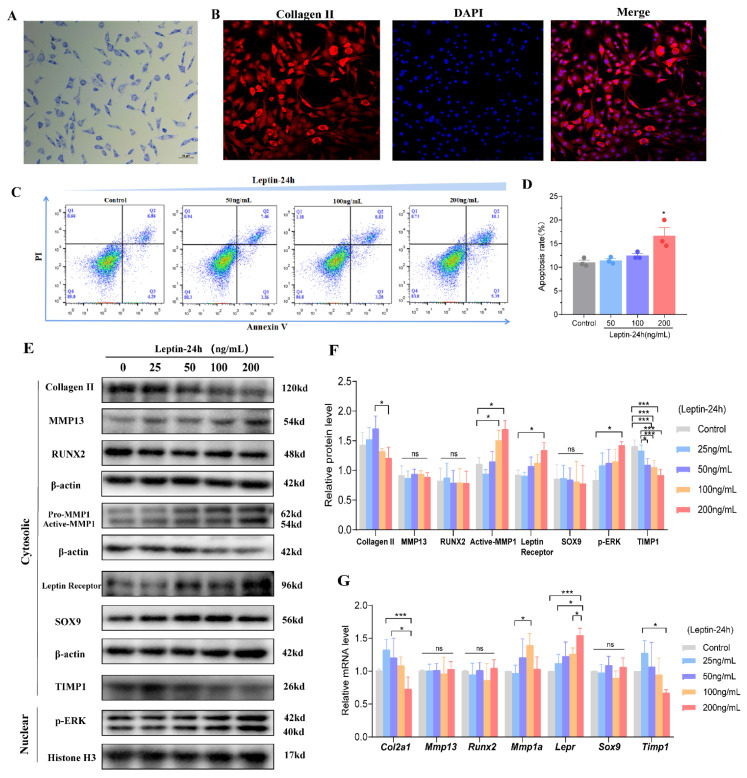
Expression of key cartilage-related factors under different doses of leptin in vitro. (**A**) Toluidine blue staining of chondrocytes (×200 magnification). (**B**) Representative collagen II immunofluorescent staining (×200 magnification). (**C**) Detection of apoptosis by flow cytometry (*n* = 3, chondrocytes obtained from three separate repeated experiments). (**D**) Statistical analysis of apoptosis rate. (**E**) Representative western blot bands of the expression of p-ERK in the nucleus and collagen II, MMP13, RUNX2, MMP1, Leptin Receptor, SOX9, and TIMP1 in the cell cytoplasm induced by leptin in vitro (*n* = 3, chondrocytes obtained from three separate repeated experiments). (**F**) Statistical analysis of the relative protein levels. (**G**) Relative mRNA expression of cartilage-related factors under different doses of leptin treatment for 24 h in vitro (*n* = 4, chondrocytes obtained from four separate repeated experiments). Results are expressed as mean ± SD. ns not significant, * *p* < 0.05, *** *p* < 0.001.

First, the chondrocytes were identified as described previously [26]; toluidine blue staining and collagen II immunofluorescence staining proved successful for in vitro chondrocyte extraction (Figure 5A,B). Further, Annexin V and PI double-stained experiments showed that high dose leptin caused chondrocyte apoptosis in vitro experiments (Figure 5C,D, *p* < 0.05). Moreover, the expression of the chondrocyte nuclear protein p-ERK increased with leptin dose, verifying the activation of MAPK/ERK signaling pathway under leptin action, the levels of the collagen II mRNA and protein levels first increased and then decreased during leptin treatment (Figure 5E–G, *p* < 0.05). In addition, a trend of increasing leptin receptor, MMP1 mRNA, and protein levels in chondrocytes with increasing leptin dose in vitro existed (Figure 5E–G, *p* < 0.05). *Timp1* mRNA showed a trend of increasing and then decreasing with the increasing leptin dose (Figure 5G, *p* < 0.05), while TIMP1 protein showed decreased expression under the high dose of leptin (Figure 5E,F, *p* < 0.05). However, we observed no significant alterations in chondrocyte RUNX2, SOX9, and MMP13 expression with different doses of leptin (Figure 5E–G, all *p* > 0.05).

### 3.5. Inhibition of MAPK/ERK Signaling Pathway Altered the Expression of Collagen II, MMP1, and TIMP1 in Mouse Chondrocytes

**Figure 6:**
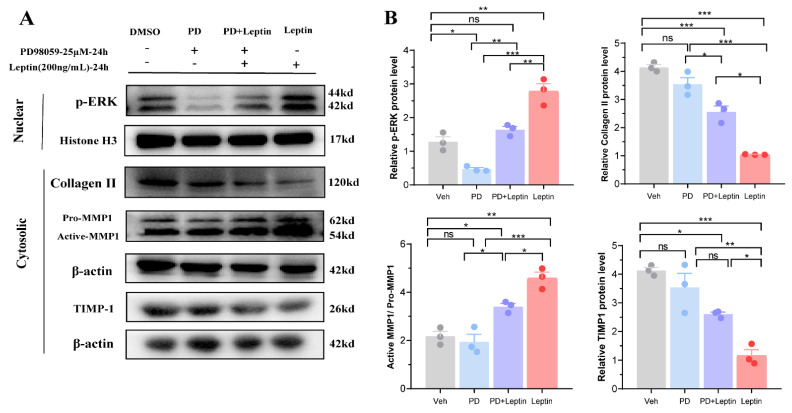
Inhibition of MAPK/ERK signaling pathway altered the expression of collagen II, MMP1, and TIMP1 in vitro. (**A**) Representative western blot bands of the expression of collagen II, MMP1, and TIMP1 in the cell cytoplasm and p-ERK in the nucleus induced by leptin, *PD98059*, or dimethyl sulfoxide (DMSO) (carrier for *PD98059*) in vitro (*n* = 3, chondrocytes obtained from three separate repeated experiments). (**B**) Statistical analysis of relative protein levels. Results are expressed as mean ± SD. ns not significant, * *p* < 0.05, ** *p* < 0.01, and *** *p* < 0.001.

Given the dose-dependent changes in collagen II, MMP1, and TIMP1 expression with the increasing leptin dose, we hypothesized that it was closely related to the leptin-activated MAPK/ERK signaling pathway. We used the MAPK/ERK signaling pathway inhibitor *PD98059* to observe leptin-mediated changes in the expression of collagen II, MMP1, and TIMP1 in chondrocytes. Further, we found that *PD98059* reversed the leptin-induced decrease in chondrocyte collagen II, TIMP1, and MMP1 expression after the addition of *PD98059* (Figure 6A,B, all *p* < 0.05). The results indicated that the MAPK/ERK signaling pathway mediated leptin-induced reduction of collagen II in mouse chondrocytes and that *PD980059* reversed the leptin-induced expression of collagen II, MMP1, and TIMP1 in chondrocytes.

### 3.6. Reduced Expression of Proteoglycan and Collagen II in Knee Cartilage of Male Mice from the Maternal Obesity Group Persisted into Adulthood

**Figure 7:**
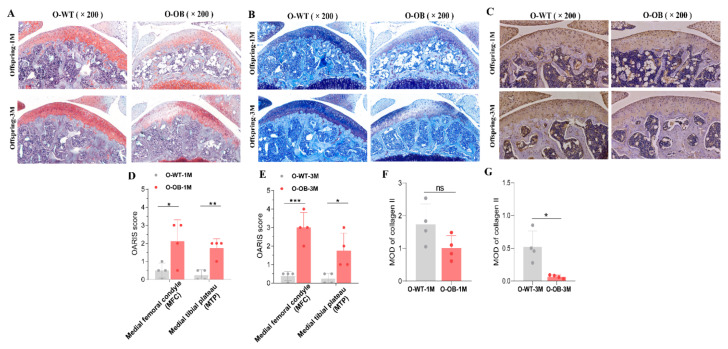
Results of pathological alterations in knee cartilage of 1- and 3-month-old male mice. (**A**) Representative images of knee cartilage stained with Safranin O/Fast Green and (**B**) toluidine blue in control versus obese offspring (×200 magnification). (**C**) Representative images of IHC of collagen II in knee cartilage of control versus obese offspring of 1- and 3-month-old mice (×200 magnification). (**D**) Statistical results of the OARIS score for 1- and (**E**) 3-month-old mice cartilage. (**F**,**G**) Statistical results of IHC-stained collagen II in each group. 1M: 1-month-old male mice from the control or obese mothers. 3M: 3-month-old male mice from the control or obese mothers. *n* = 4 represents four male mice in each group from four litters of different mothers. Results are expressed as mean ± SD. ns not significant, * *p* < 0.05, ** *p* < 0.01, and *** *p* < 0.001.

We further explored pathological alterations in knee cartilage of 1- and 3-month-old mice in the control and obese groups, and the results are shown in Figure 7. Safranin O/Fast Green (Figure 7A) and toluidine blue (Figure 7B) showed that the local proteoglycan content of knee cartilage was lower in the obese offspring than in the control offspring, and the OARIS scores were lower than in the control offspring (Figure 7D,E, both *p* < 0.05), whether the offspring were 1- or 3-month-old mice. A decreasing trend in collagen II expression was found in the knee cartilage of 1-month-old mice from the maternal obesity group (O-OB-1M) relative to the control group (O-WT-1M), with no statistically significant difference (Figure 7F, *p* > 0.05). However, a lack of significant chondrocyte hierarchy and a decrease in the number of chondrocytes were observed in the cartilage of 1-month-old mice from the obesity group (Figure 7C). At 3 months of age, collagen II expression and browning of the cartilage matrix were reduced in knee chondrocytes (Figure 7C,G, *p* < 0.05) in male obese offspring (O-OB-3M) relative to controls (O-WT-3M), suggesting that the reduction of collagen II expression and the decreased cartilage proteoglycans in male obese offspring was not a transient change and might even persist into adulthood.

## 4. Discussion

The early healthy development of cartilage is of vital importance in the proper development of long bones and skeleton. For example, exposure of cartilage to fungal toxins in early life can lead to the Kashin-Beck disease (KBD) in children, resulting in irreversible lifelong disability [28]. The present study showed that maternal obesity led to abnormal expression of collagen II in knee cartilage of male neonatal mice and then demonstrated that high leptin expression in obese male neonatal mice was closely related to the expression of key cartilage factors. Finally, high leptin expression was shown to mediate MAPK/ERK signaling pathway activation, leading to abnormal expression of key cartilage genes and proteins in offspring male mice from the maternal obesity group, resulting in chondrocyte damage and reduced collagen II expressions.

Collagen II is classically considered to be an essential collagen component in articular cartilage and plays a key role in the development and maturation of chondrocytes [29], accounting for 90% of all collagen in cartilage [30]. Uneven distribution of matrix was observed on the cartilage surface of the knee joint in male neonatal mice from the maternal obesity group. Together with the results of Safranin O/Fast Green and toluidine blue staining, the present study showed that the coloring of knee cartilage surface was weakened, accompanied by reductions in collagen II mRNA and protein levels. Notably, the reduction in knee cartilage proteoglycan and collagen II persisted into adulthood in the obese group offspring male mice, although the offspring had returned to a normal diet after weaning. This suggested that the reduction in knee cartilage proteoglycan and collagen II in obese offspring was not an isolated effect of a high-fat diet and that a unique force of genetic programming existed from early in life.

The sex-differentiated effects of early life exposure to adverse factors on offspring health have been continuously discussed in recent studies [31,32]. In the early phase of this study, we found little difference in plasma leptin levels in neonatal mice from obese mothers and collagen II expression in knee cartilage of the offspring, and compared these with controls (results can be seen in Supplementary Figure S1B,C). This seemed to be the opposite of the higher incidence of OA in women than in men in the population [33,34]. The likely reason for this is as follows; in rodents, males have higher circulating leptin levels than females, whereas in humans, females have increased circulating levels of leptin for any degree of adiposity [31,35]. In conjunction with the present study, high leptin levels may become important in explaining the chronic disease origin of OA in women in addition to the effects of estrogen. Furthermore, given that obesity is linked to systemic inflammation, the status of inflammatory factors in obese offspring by sex and whether inflammatory factors synergize with leptin in the degradation of cartilage collagen will be interesting topics for future studies exploring the sex-differentiated effects of adverse intrauterine exposure on the origin of cartilage development.

In this study, we found abnormal expression of cartilage-related regulators in male neonatal mice from obese mothers. SOX9 and RUNX2 mRNA and protein levels were downregulated in the cartilage of male neonatal mice of obese mothers, accompanied by increased MMP1 and decreased TIMP1 mRNA and protein levels, compared with those in offspring neonatal mice on a normal diet. However, no difference in MMP13 expression existed between the two groups. The evidence previously found was as follows; SOX9 was found to directly regulate collagen II, and SOX9 deficiency prompted growth-plate chondrocytes at all stages to swiftly reach a terminal/dedifferentiated stage [36,37]. RUNX2 was associated with catabolic phenotypes observed in OA in addition to its important role in the differentiation of cartilage hypertrophy [38]. The collagenolytic MMPs were all produced by chondrocytes as well as by cells in the synovium [39]. The balance between MMPs and TIMPs was tightly controlled in healthy joints, and an excess of MMPs over locally available TIMPs could lead to excessive degradation of the ECM [40]. In brief, the alterations in key cartilage transcription factors, as well as the imbalance between MMP1 and TIMP1, might be important in causing reduced cartilage matrix collagen II expression in this study.

Furthermore, we were interested in which factors might mediate the reduction of collagen II and the alteration of cartilage-related regulators in cartilage in male neonatal mice of obese mothers. Subsequently, we found increased levels of leptin in the plasma of newborn mice of an obese mother. The chondrocyte expression of leptin receptors [41] suggested that leptin might affect chondrocytes via leptin receptors. At the same time, we found that *Lepr* expression at the mRNA level was closely associated with *Runx2*, *Sox9*, *Mmp1a*, and *Timp1* expression. Studies have shown that leptin acts as a pro-inflammatory adipokine with a catabolic role in cartilage metabolism via the upregulation of proteolytic enzymes [42,43]. In this study, we found that in vitro stimulation of chondrocytes with different concentrations of leptin resulted in a trend of increasing and then decreasing cellular collagen II expression, which seemed to suggest a possible bidirectional nature of the leptin regulation of chondrocytes.

The MAPK signaling pathway has been reported as one of the major signaling pathways mediated by leptin and leptin receptors [43]. In the present study, the level of ERK protein phosphorylation in the cartilage MAPK signaling pathway was enhanced in newborn male mice from obese mothers, whereas the activation levels of p-p38 and p-JNK in the MAPK signaling pathway were less altered compared with ERK phosphorylation levels. Several studies have reported important roles of ERK signaling pathways in cartilage and cartilage-related diseases, including the role of the MAPK/ERK pathway in controlling chondrocyte hypertrophy [44]. Another study reported that ERK signaling was one of the most important contributors to the pathogenesis of KBD [45]. Considering this evidence, we hypothesized that the activation of the cartilage leptin and leptin receptor signaling pathway-mediated ERK signaling pathway in male neonatal mice from obese mothers was likely to be an important factor in the decline of cartilage collagen II.

**Figure 8:**
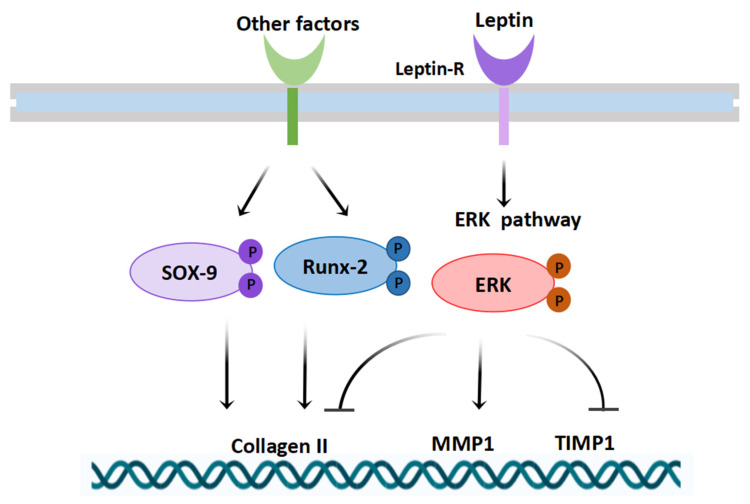
Possible mechanism of maternal obesity-induced cartilage damage in the newborn male mice.

Finally, to demonstrate whether the relationship between leptin and key factors of cartilage was mediated by the MAPK/ERK signaling pathway, we performed primary cultures of mouse chondrocytes in vitro and found that leptin dose showed a dose-response relationship with chondrocyte expression of phosphorylated ERK, MMP1, and TIMP1. However, SOX9 and RUNX2 did not seem to show a significant increase or decrease in response to leptin in vitro. Furthermore, the inhibition of the ERK signaling pathway resulted in an improved increase in chondrocyte collagen II content, accompanied by a decrease in MMP1 protein expression levels and an increase in TIMP1 protein levels in vitro. It is presumed that other key factors may be present in male offspring in the maternally obese group to regulate the expression of SOX9 and RUNX2 in cartilage, rather than leptin alone. The resulting conjectural map of the mechanism of maternal obesity-induced cartilage matrix damage in neonatal male mice is shown in Figure 8.

The present study had some limitations. For example, whether leptin-activated phosphorylated ERK was directly involved in the regulation of collagen II, MMP1, and TIMP1 transcription factors and, thus, affected the expression of these factors, or whether phosphorylated ERK activated other signaling pathways and, thus, regulated the factors needs further investigation.

In conclusion, our study was novel in demonstrating that collagen II expression decreased in the cartilage matrix of male neonatal mice from obese mothers, which was closely associated to high leptin expression in the offspring of the obese group, and that leptin mediated MAPK/ERK signaling pathway activation, affecting the imbalance of cartilage MMP1 and TIMP1 expression, finally leading to abnormal cartilage collagen II.

## Figures and Tables

**Table 1:** Information of primers for real-time RT-PCR.

mRNA	Sequence (5′-3′)
*Col2a1*	F:GCTGGTGAAGAAGGCAAACGAG R:CCATCTTGACCTGGGAATCCAC
*Lepr*	F: GGATGTGCGTTGGAGGACTATG R: GAAATACGCCAGTTCCCGACAG
*Sox9*	F:CACACGTCAAGCGACCCATGAA R: TCTTCTCGCTCTCGTTCAGCAG
*Runx2*	F:CCTGAACTCTGCACCAAGTCCT R:TCATCTGGCTCAGATAGGAGGG
*Mmp13*	F: GATGACCTGTCTGAGGAAGACC R: GCATTTCTCGGAGCCTGTCAAC
*Mmp1a*	F:AGGAAGGCGATATTGTGCTCTCC R:TGGCTGGAAAGTGTGAGCAAGC
*Timp1*	F: TCTTGGTTCCCTGGCGTACTCT R:GTGAGTGTCACTCTCCAGTTTGC
*Gapdh*	F:CATCACTGCCACCCAGAAGACTG R: ATGCCAGTGAGCTTCCCGTTCAG

## Data Availability

Not applicable.

## Supplementary Materials

The following supporting information can be downloaded at: https://www.mdpi.com/article/10.3390/biom12030477/s1, Figure S1: Results of TUNEL staining of knee cartilage in neonatal 7-day male mice from control and obese offspring, and cartilage collagen II and plasma leptin expression in neonatal 7-day female mice from control and obese offspring; Figure S2: Full view of the knee joint in obese and normal offspring of newborn 7th-day male mice; Table S1: Statistical description and analysis of Figure 1; Table S2: *p*-values of the correlation coefficients for Figure 4C.
