# Supplementary material for: Effects of the Leptin-Mediated MAPK/ERK Signaling Pathway on Collagen II Expression in Knee Cartilage of Newborn Male Mice from Obese Maternal Offspring
Source: Biomolecules. 2022 Mar 21;12(3):477. doi: 10.3390/biom12030477 (PMC8946789; doi:10.3390/biom12030477)
Supplement: Supplementary file 1 [file biomolecules-12-00477-s001.zip › biomolecules-1613110-supplementary.pdf]

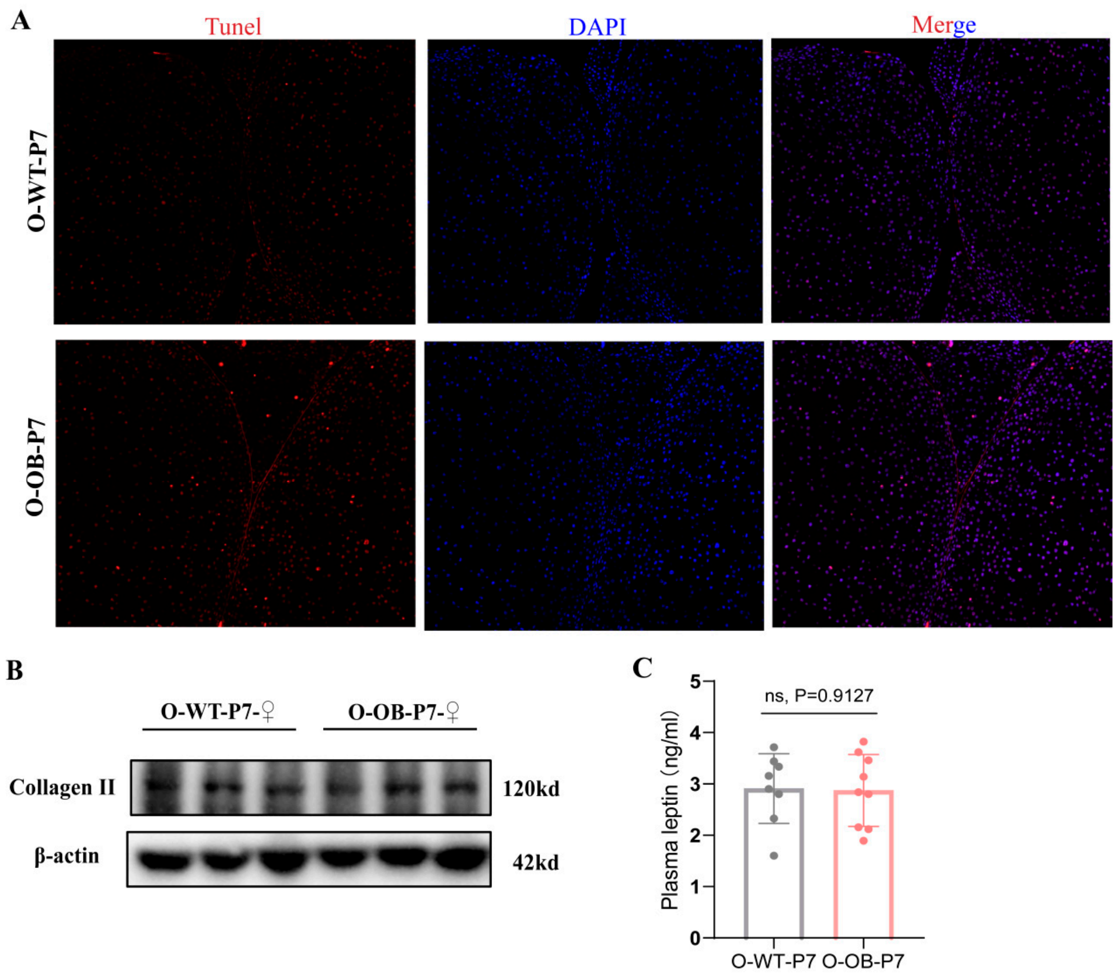

Figure S1. Results of TUNEL staining of knee cartilage in neonatal 7-day male mice from control and obese offspring, and cartilage collagen II and plasma leptin expression in neonatal 7-day female mice from control and obese offspring. **(A)** Representative images of TUNEL staining of knee cartilage in knee cartilage 7-day male mice offspring in control versus obese group (×200 magnification). **(B)** Representative western blot bands of the expression of collagen II in knee cartilage 7-day female mice offspring in control versus obese group. **(C)** Plasma leptin level of 7-day female mice offspring in control versus obesity groups.

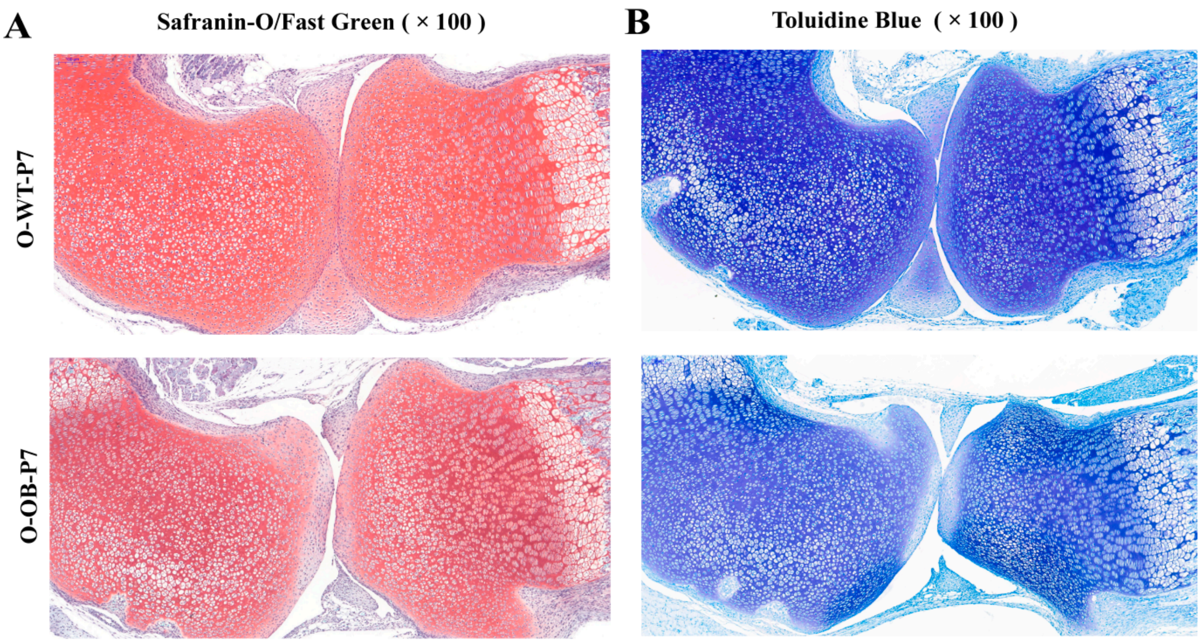

Figure S2. Full view of the knee joint in obese and normal offspring of newborn 7th-day male mice. **(A)** Representative images of knee cartilage stained with Safranin O/fast green and **(B)** toluidine blue in control versus obese offspring (×200 magnification).

Table S1. Statistical description and analysis of Figure 1.

	**WT-mother (n=8)**	**OB-mother (n=8)**	**t**	**P**
Weight (g)-4-week-old	16.66±0.82	16.47±1.02	0.42	0.682
Weight (g)-12-week-old	19.26±1.04	23.76±1.52	-6.93	<0.001
	O-WT-P7 (n=25)	O-OB-P7 (n=19)	t	P
Weight (g)	3.38±0.29	2.76±0.56	4.36	<0.001
Length (cm)	3.99±0.12	3.77±0.20	4.29	<0.001
BMI (g/cm^2)	0.21±0.02	0.19±0.03	2.66	0.011

Table S2. *P*-values of the correlation coefficients for Figure 4C.

	LEPR	***COL2A1***	***RUNX2***	***SOX9***	***MMP1a***	***TIMP1***
TIMP1	0.001	0.001	0.003	0.003	0.079	-
MMP1a	0.028	0.013	0.007	0.031	-	0.079
SOX9	<0.001	<0.001	0.001	-	0.031	0.003
RUNX2	<0.001	<0.001	-	0.001	0.007	0.003
COL2A1	<0.001	-	<0.001	<0.001	0.013	0.001
LEPR	-	<0.001	<0.001	<0.001	0.028	0.001
